# Surface Morphology Analysis of Knit Structure-Based Triboelectric Nanogenerator for Enhancing the Transfer Charge

**DOI:** 10.1186/s11671-020-03401-1

**Published:** 2020-09-22

**Authors:** Li Niu, Xuhong Miao, Yutian Li, Xinkai Xie, Zhen Wen, Gaoming Jiang

**Affiliations:** 1 Engineering Research Center for Knitting Technology, Ministry of Education, Jiangnan University, Wuxi, China; 2 School of Textiles and Clothing, Jiangnan University, Wuxi, China; 3 Institute of Functional Nano and Soft Materials (FUNSOM), Jiangsu Key Laboratory for Carbon-Based Functional Materials and Devices, Soochow University, Suzhou, China

**Keywords:** Knit structure, Surface morphology, Fractal theory, Transfer charge, Biomechanical harvest

## Abstract

Harvesting waste biomechanical energy has provided a promising approach to improve the power supplement of wearable devices for extending usage life. Surface morphology is a significant factor for enhancing output performance of triboelectric nanogenerator; however, there is a limitation for evaluating the morphology of the surface and its impact on power generation. To evaluate the relationship between the surface morphology and transfer charge, there is a mathematical theory that is the fractal geometry theory that has been proposed to analyze the characteristic of irregular surface morphology. This theory provided a good understanding of the contact area and roughness of the surface. We have designed three categories of knit structures with cord appearance by using a flat knitting machine and analyzed their surface characteristics. Meanwhile, the geometric structures can be demonstrated through the fractal dimension for evaluating the generated output performance during contacting and separation. The present research exhibits that, with the increasing number of knitted units, the triboelectric power-generation performance continued to reduce due to the available contact area decreasing. After calculating the fractal dimension of different knit structures, the m*n rib structures show the high transfer charge when the fractal dimension is close to number one, especially the fractal dimension of the 1*1 rib structure that can reach 0.99. The fractal theory can be further used as an approach to evaluate the influence on the output performance of irregular surface morphology, unrelated to the uniform convex unit distraction. The result of this research also demonstrated the feasibility of a knitted-based triboelectric nanogenerator in scavenging biomechanical energy for powering portable electronics integrated into garments.

## Introduction

Advanced intelligence techniques have swept the global world and have brought out some novel flexible smart wearable devices, such as health tracking sensors [1, 2], gesture-detecting devices [3–6], electronic skins (E-skins) [7, 8], flexible circuits [9, 10], and optical fiber wearables [11, 12]. However, with disadvantages of mass weight, low conversion efficiency, serious environmental pollution, and short battery life, the power supplement is the enormous limitation for the development of electronics. Since the first triboelectric nanogenerator (TENG) has been developed successfully in 2012 [13], based on the characteristic of small scale, lightweight, various materials, safe, environmental virtues [14], and high efficiency, it has provided a promising and effective strategy to address above straits. Along with the rapid advent of TENGs working through a coupled effect of contact electrification and electrostatic induction [15], it has been conformed as one desirable approach to gain mechanical power [16, 17] from our surrounding especially by harvesting low-frequency and irregular movements (including wind [18, 19], waterdrop and human motion, biomechanical energy, etc. [20–22]), realizing data transmissions [23–25] and power supplement in the Internet of Things (IoT) [26]. For wearable devices, textiles are regarded as the best substrate, due to its structural retention and fatigue resistance, soft, integration, and high porosity. To date, an integration of a triboelectric nanogenerator and traditional textile [27–33] is one of the promising candidate for human-oriented wearable devices, such as self-powered flexible sensors [34], wearable energy harvesters, and textile-based energy storage systems. It is also endowed conventional textiles with functionality, intelligence, and highadditionalvalue. These electronic devices based on the textile that are satisfied with the requirement of lightweight, inexpensive, comfortable, breathable, portable, long-lasting, and washable for routine usage. In addition, it is facile to make textile with variable colors and abundant pattern designs which represent attractiveness for intelligent textiles. Especially, knit textiles with small strain and large deformation are sensitive to signal generation thus are ideal to be used for flexible sensors, overcoming movement resistance, and reducing energy loss [35]. Additionally, frictions and deformations of knit textile are common phenomena that are a thrilling opinion for constructing a triboelectric nanogenerator.

As we all know, surface-morphology modification is a significant approach to enhance output performance of TENGs [36–39]. Most are purposed on increasing the available contact area and roughness of the surface. There are two primary methods which change the surface morphology, one being surface etching, the other being surface replication. However, the use of highly expensive, limited treatment area and multi-step manufacturing technique to generate surface appearance is difficult for industrial production. Herein, Li et al. [40] investigated a polydimethylsiloxane (PDMS) film with surface microstructures peeled off from the sandpaper, which was a one-process and low-cost method to prepare the difference roughness of the surface. The experimental results showed the generated maximum output of 46.52 V under the roughness class of 3000 detected by a 3D optical surface profile. Besides, too many microstructures can decrease severely the effective contact surface and result in the reduction of the ability of power performance. The size of TENGs was limited by the sandpaper area, which led to increase the fabrication cost. Nowadays, textile structures are receiving increasing attention due to formation of abundant surface appearances [38] without the complex fabrication procession and high cost. For fully understanding textile surface appearances, some factors need to be considered in terms of unique components and structure features, including the thread outlook, textile physical parameters, and knit structures. Then, Kwak et al. [41] investigated the contact area of three structures (including plain-, double-, and rib-fabric structures) during stretching and discussed the contribution for enhancing the potential. It was worth that rib-fabric can be strained up to 30%, enlarging contact area to 180 cm^2^. Depending on the middle region existing, rib fabric can be stretched largely, which can obtain a higher potential for increasing contact area. As the primary element of the textile structure, the characteristic of loops was analyzed that was also the significant factor for influencing surface appearance. Huang et al. [42] made a focus on the effect of basic parameters of textile (including loop legs, loop sinkers, and textile density) for confirming the difference on the output performance. The large stitch density fabric-based triboelectric nanogenerator could generate higher electric energy with a maximum peak power density of 203 mW m^−2^ at 80 MΩ, which makes a larger effective contact area. The result exhibited that the surface morphologies of various fabric structures had an influence on the electrical-output ability. In order to harvest much more energy for extending lifetime, 3D double-faced interlock stitch textiles [43] were knitted by double-needle bed flat, which exhibited the same output performance on front and back side. In addition, the TENGs based on the three-dimensional textile structure could generate a high-power density of 3.4 mW m^−2^ at the external resistance of 200 MΩ, demonstrating that the capacity of energy harvesting has been improved. However, the abovementioned surface appearances have little depiction on the geometry shape of the surface, and factors about generated transfer charge are still suffering from lack of specific explanations. There is no universal manner that can characterize surface appearance, which needs to find an evaluation of irregular morphology. Therefore, that is the limitation for fully understanding the transfer charge on the triboelectric nanogenerator currently.

The purpose of surface analyzation is to characterize textiles’ geometric structures, which may be tested in two approaches of contact method and optical method [44]. The contact method can describe surface morphology well, but the time needed is much longer, and the needle leaves a trace on the surface. Compared to the contact method, with the benefits of short measurement time, low harness surface, and easy detection, the optical method has been used for detecting surface roughness. However, the false gaps and high level of noise may reduce the judgment of the real surface morphology.

The mathematical tool is a theory analysis that can be used to quantify the extent of surface roughness. It is a novel approach to evaluate the irregular surface. With such an uneven surface, the conventional mathematical method of Euclidean geometry cannot be used because it is really hard to judge the quantitative geometry dimension and measurement accuracy, such as length of segment and weight of the object. However, fractal geometry, an approach named by Mandelbrot for describing irregular structures, has been provided to solve the issue and define the irregularity in nature [45], such as the physical properties of foams [46] and evaluation for fabric smoothness [47]. Almost all the rough surfaces can be divided into some self-similar parts which can be depicted by a non-integral dimension, named fractal dimension (*D*_f_). Based on the various geometric surfaces, the value of *D*_f_ needs to be considered and analyzed that has an effect on the roughness and efficient contact area in the design of a triboelectric nanogenerator, optimizing the capacity of converting human motions into electrical.

Herein, in this work, we present the various surface morphologies based on knit structures that are adopted as one of the dielectric layers. The knit-textile-based TENG was fabricated by using commercial threads and industrial knitting machine, which can realize the large-scale production and practical applications. To imitate the flapping hand movement, TENGs are designed in the contact-separate working mode (CS) which is the simplest working mechanism. The knit structures are formed in two kinds of approach, including structured- and shaped-based convex-concave surface morphology. Due to the diversity of knit structures, the resultant surface appearances can be systematically investigated and analyzed for confirming the relationship between surface morphology and knit structures. The D_f_ of every fabric can be calculated through the appropriate fractal principle, evaluating the roughness of the fabric surface. The maximum transfer charge of surface appearance in 1*1 rib can reach up to 91.66 nC by flapping and releasing motion, which obtain the fractal dimension of 0.99. And an interesting phenomenon exhibits that with the value of *D*_f_ closing to the number one, the transfer charge can be higher. Finally, using the fractal theory and knit structures can provide an effective method for quantity evaluating the transfer charge and are expected to be of help to design the knit-textile-based TENGs with more efficiency, industrial production, and inexpensive cost.

## Materials and Methods

### Materials

The nylon yarns (dtex 600, AnTong KeJia Textile fiber products Co., Ltd.) which were commonly available knitted into two kinds of rib textiles and convex fabrics with a gage of 15 (needle/inch) on the whole garment machine (SHIMA Seiki Co., Japan). The film of polytetrafluoroethylene (PTFE) with a thickness of 0.05 cm (Chenqi Electrical Technique Co. Ltd.) is used. The bent and twisted electrode is commercial copper foil (Shenzhen Biaozhitape Co. Ltd) with a thickness of 0.06 mm pasting on the back of knitted-textile for transferring polarized charge.

### Fabrication of the Knitted Fabrics and Knitted-Textile-Based Triboelectric Nanogenerator

The weft technique as the representative knitting method can easily endow fabrics with high stretchability [48], low cost, and esthetic performance. With the advantages of position knitting, the power textiles can be integrated into clothing without additional sewing techniques. There are ten convex-concave textures designed that are depicted in Table 1. To demonstrate the relationship between surface morphology and transfer charge, longitude and transverse cords are knitted on the surface of textile. So ten different textures are depicted in Table 1, wherein the first seven samples are exhibited longitudinal cord on the surface, and the surface appearances of no. 8, no. 9, and no. 10 are transverse convex. Here, the structures are knitted in by a computerized flat-knitting machine which is suitable for high-efficient industrial process, and the textiles are able to tailor the custom scale. Via the own design system, fabrics can be designed quickly and prepared facilely, especially for designing intricate patterns. All fabrics need to be left for 24 h with the standard atmospheric condition for relaxing fabric to the state of size stable, which are purposed on declining the influence of relaxation shrinkage and enhancing the result of testing accuracy. Then, the same size of conductive tape was pasted on the back of textiles. Based on the highly polarizable nanoparticles, the film made of PTFE was adopted as the other dielectric materials. The film is still adhered to a piece of copper foil, transferring electron migration. As for the CS, conducting wires have been connected to two friction models, which move in the vertical direction. Then the CS-based textile TENGs have been fabricated.

**Table 1:** Summary of sample

Classification	Sample no.	Knit structure
m*n rib	no. 1	1*1 rib
	no. 2	2*2 rib
	no. 3	3*3 rib
	no. 4	4*4 rib
2*m rib	no. 5	2*1 rib
	no. 2	2*2 rib
	no. 6	2*3 rib
	no. 7	2*4 rib
n	no. 8	Four needles horizontally cord
	no. 9	Five needles horizontally cord
	no. 10	Six needles horizontally cord

### Fractal Characters of Knitted Fabrics

Not all natural objects are of incomplete regular shape and boundary, including coastline, snowflake, cloud, and leaf. Therefore, the fractal dimension is used to describe the uneven morphology generated by different methods, which is an effective method identified in many research works. There are several formulations defined as fractal dimension, including the Hausdorff dimension, the counting box dimension, and similar dimension et al., which is the crucial parameter to quantify the style of the surface. The typical fractal dimension was the Kohn curve like a snowflake, which was first presented in 1904. The area bounded by three self-similarities with infinite is restricted, named Kohn curves, which fractal dimension is 1.2618. Generally, the fractal dimension can be calculated by scale a, which indicates the length, width, and area. The following formula can present the relationship:

1-1 $$ F\left(\mathrm{a}\right)\approx {a}^{D_f} $$

where *D*_f_ is the fractal dimension which is exhibited in the slope of a log-log plot.

The uneven surface fractal dimension, *D*_f_, can be determined in an approach of Hausdorff dimension that is based on the relative size analysis of a similar unit. As the factor of forming cord surface, the convex district which includes several micro-convex structure units with different edges and number can be expressed as:

1-2 $$ M={N}^{D_f} $$

where *M* is the number of the convex unit, *N* is the repeated multiple self-similar units that is the length of convex units to the length of whole samples, and *D*_f_ is the raised structures’ fractal dimension. The equation is a model that can be used to predict the surface morphology, so:

1-3 $$ {D}_f=\raisebox{1ex}{$\log M$}\!\left/ \!\raisebox{-1ex}{$\log N$}\right. $$

### Characterization

The Dino-lite edge digital microscope (AnMo electronic corporation) was used to measure the density of knitted fabrics from photo images. The electrical signals of knitted-fabric triboelectric nanogenerator while contacting and separating mode were operated by a self-assemble liner motor and an electrometer (Keithley 6514 system) based on the LabVIEW system.

## Results and Discussion

In order to confirm friction materials, the triboelectric order [49] is the significant reference, which quantified the triboelectric polarization of different common materials. The triboelectric order presents that one side shows gaining charge capacity and the other side owns a high ability to lose electrons, which have been defined as the fundamental material performance. To obtain the outstanding output performance, a couple of materials are selected that need to be attributed to the triboelectric series with a considerable distance, increasing the potentialdifference. Herein, one is the commercial, low-cost, excellent abrasion resistance and highly positively charged tendency (nylon) and the other one shows negatively charged tendency (PTFE). In this work, we selected the PTFE membrane without any treatments on the surface. Herein, the only factor is knit structures that can be analyzed by the performance of transferring charge. Another critical element is the electrode material that is copper foil with high flexibility, which can be pasted directly, that is a simple and one-step fabrication process. Compared to the precious metal of silver and gold, the price of copper foil is inexpensive and can be used to fabricate the economical products. So copper has been widely applied as flexible circuits and electrodes in the design of smart devices.

**Fig. 1:**
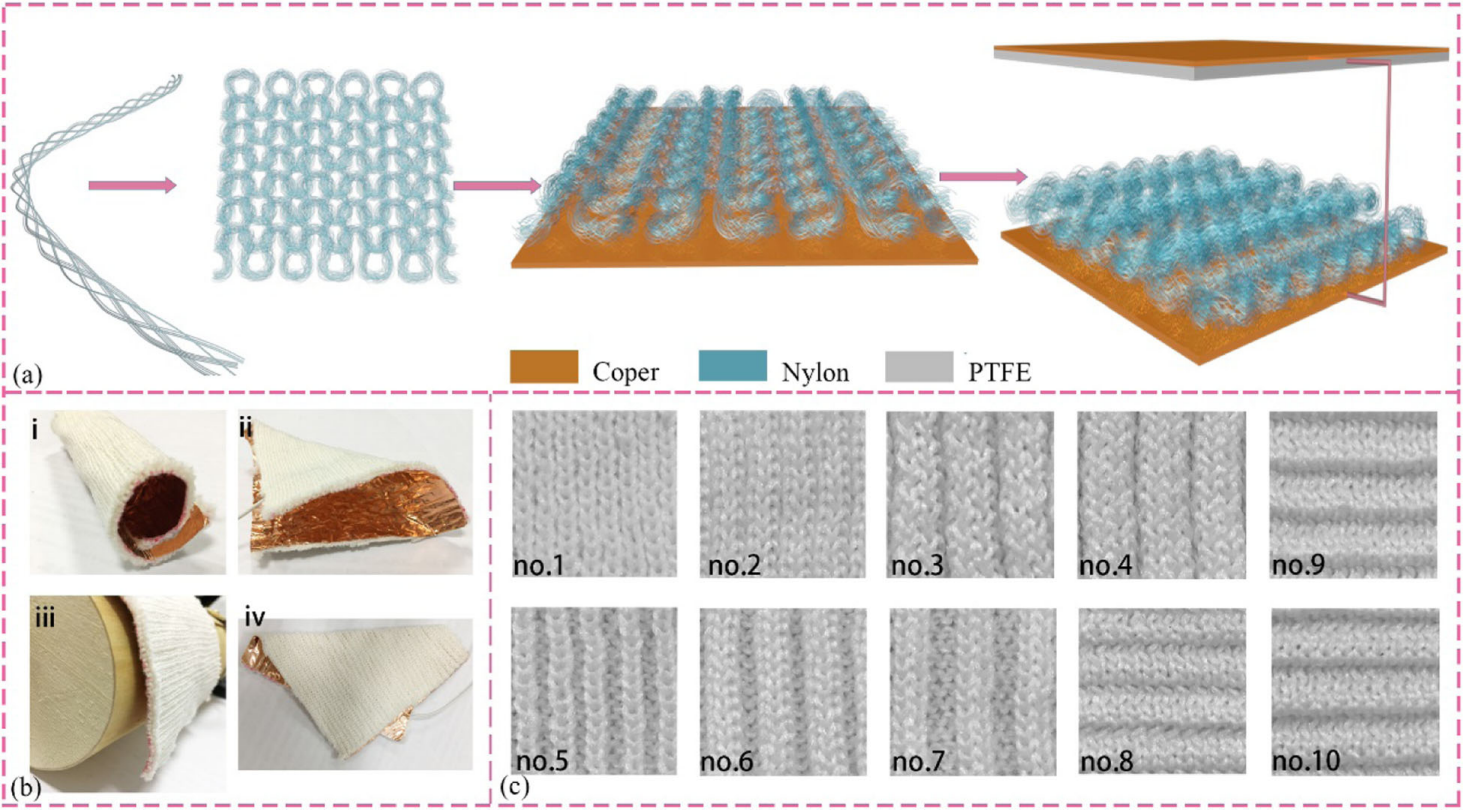
Schematic preparation, characteristic of KNG, and knit structure. **a** Fabrication process of KNG. **b** Images of KNG under various deformation. i, crimped; ii, bent; iii, draped; iv, folded. **c** All of the fabricated knit structures, from number 1 to 10

At present, there are four universal working mode-operated TENGs corresponding to the different electrode structures and movements. With advantages of facile fabrication, the abundant material selection, reciprocating vertical direction movements, the CS TENGs are the first deeply investigated that have the potential ability for harvesting some biomechanical energy, such as flapping hands, walking, and running. Here, in order to investigate the influence principle of the surface structures, the triboelectric nanogenerators based on knitted textile (KNGs) have been designed, corresponding to the contact and separation between nylon fabric and PTFE film. The process of assembling the triboelectric nanogenerator is presented in Fig. 1a, consisting of knitted fabrics, PTFE membrane, and copper foil. The versatility of flexible knitted fabric in terms of its capacity to crimping (Fig. 1 bi), bending (Fig. 1 bii), draping (Fig. 1biii), and folding (Fig. 1biv) in any direction is tailored in various scales depicted in Fig. 1b. The KNGs can be designed based on the requirement of application position and the esthetics of clothing. The diversity of knit structures has been knitted with different surface appearances, and then these photographs of the textile surface have been shown in Fig. 1c.

**Fig. 2:**
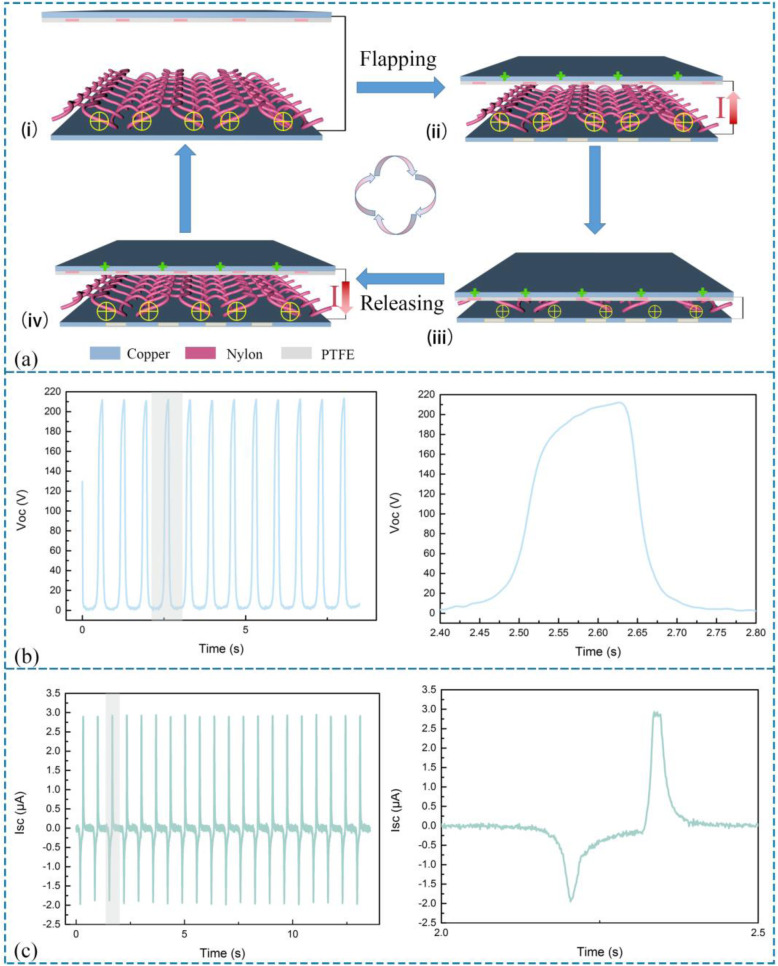
Electrical power working mechanism and the output performance of KNG. **a** Operation mechanism of KNG using nylon fabric contact with PTFE member. **b** Voc of KNG and enlarged image for one cycle. **c** Isc of KNG and enlarged image of one cycle

The operation mechanism of the KNGs is simply presented in Fig. 2a. To measure the transfer charge, the maximum distance and the frequency of movements of the linear motor are set as 10 cm and at 0.3 Hz for simulating flapping hands motions, respectively. As for common monitorization, the open-circuit voltage (Voc), short-circuit current (Isc), and transfer charge (Qsc) are measured by a mechanical linear motor. In the original state (Fig. 2 ai), the nylon textile produced positive charges and PTFE film was charged with negative charges because of the electrostatic induction and conservation of charges. When the device was pressed (Fig. 2 aii), a shrinkage of the gap between the both contact surfaces will lead to the positive charge accumulating in the electrode pasted on the PTFE. The electrons flow from the external circuit for balancing the potential difference. It was worth noticing that the equivalent amount of electrons can be maintained on the surface of the contact area because both dielectric materials are insulators (Fig. 2 aiii). As the PTFE moves back (Fig. 2aiv), the process reversed and the electric will be obtained balance once again between nylon textile and PTFE, reflecting the neutralization of charges. Consequently, the electrons will flow back for electrical potential differences. In this situation, the KNGs could generate Isc and Voc, which have a characteristic of periodical change, shown in Fig. 2 b and c. In Fig. 2b and c, the inset is an enlarged graph which is described in one cycle.

**Fig. 3:**
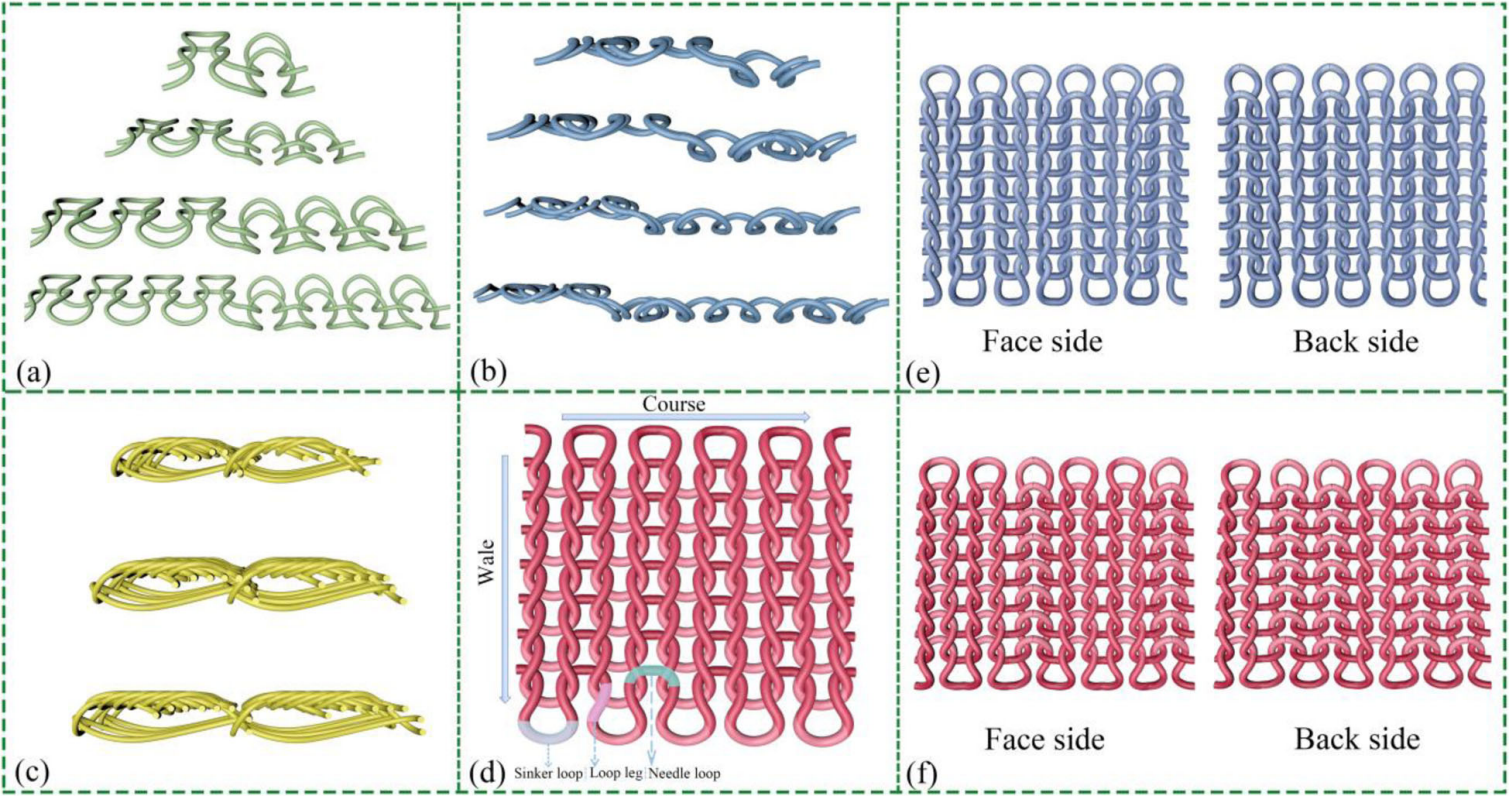
Schematic characteristics and components of knit structures. **a** Characteristics of m*n rib. **b** Characteristics of 2*m rib. **c** Characteristics of some needle horizontal cord. **d** Knitting-technique nomenclature. **e** Image of the face side and back side in the 1*1 rib structure. **f** Image of the face side and back side in 2* 1 rib structure

In order to fabricate convex structures on the textile surface, there are two kinds of methods used, including structure design and shape formation, as shown in Fig. 3. The structure design is dependent on the different proportion of the face loop stitches and the reverse loop stitches. The total samples are designed in seven rib types, including the type of m*n (*m* = *n* = 1, 2, 3, 4) in Fig. 3a and 2*m (*m* = 1, 2, 3, 4) shown in Fig. 3b. The rib has a vertical cord appearance due to the face loop wales that tend to move over and in front of the reverse loop wales; then, the cord maximum height can arrive at 0.2 cm. The rib of m*n (*m* = *n* = 1, 2, 3, 4) can be balanced by alternate wales of face loops on each side, so it lies flat without curl after tailoring. And both sides of the textile are the same appearance as shown in Fig. 3e. However, the different proportions of face and reverse loops in 2*m rib structures, there is a distinction surface come out, as shown in Fig. 3f. In addition, the stretching process of rib fabric is divided into two stages, including the reverse wales intermeshing on both sides until being stretched to reveal the reverse loop wales in between and then whole loops are continued to be stretched over twice as wide as an equivalent single fabric. Therefore, compared to plain fabrics, rib textiles have potential to increase the stretchable ability for harvesting flapping and stretching movements (transverse direction and longitudinal) during the contact-separation working mode. The other method for establishing raised structure is the shape deformation wherein the air layer is formed on the surface of the *n* (*n* = 4, 5, 6) textile that is illustrated in Fig. 3c. The thickness of cross-sectional area is in the range from 0.15 to 0.3 cm. The characteristic of the air layer is a prominent arch structure that can provide some space for accelerating electron separation when triggering motions. Above all mentioned, knitted textiles are designed through a computerized flat machine that can realize the knitting location accuracy, forming the whole garment and integrating smart materials into cloth perfectly. Such knitting-technique nomenclature has been marked on Fig. 3d that depicts the features of structure correctly.

**Fig. 4:**
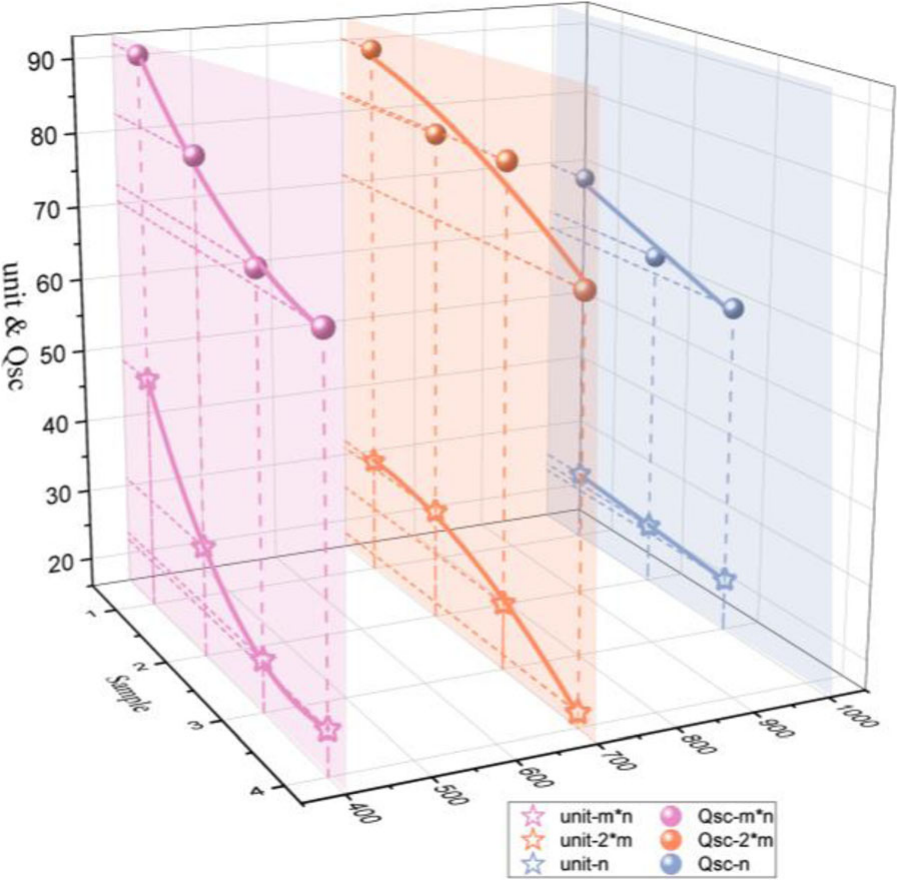
The output performance changed through the number of the convex unit

Previous works [42] demonstrated the effective contact area of face side that was much more than the back of textile; result in transfer charge was twice as high as the output performance of back side. This is because the length of needle loop was longer than the sinker loop. Therefore, to enhance the output performance and to create only one influential factor, the contact-raised structures consist of face side loops. The outputs of the KNGs depending on the number of the convex units are plotted in Fig. 4. A decreasing trend where the contact area of all experimented textiles was decreased with the number of the raised unit was formed. Also, the more significant electrical charges are in the sequential order of the 1*1 rib, 2*1 rib, and four needle shape-type structures (the first point of each line) with the values of 91.66 nC, 90.19 nC, and 69.64 nC, respectively.

Then, the knit structure with the different surface morphology in aspects of diversity wale density, number of face side unit, and structures are investigated. All of the parameters of ten kinds of knitted textiles are tested and recorded in Table 2. Notably, the course density is always constant because the cord appearance has grown along the vertical direction when analyzing sample Nos. 1–7. So, wale density as the main factor which needs to be discussed refers to the features of different knit structures. It is obvious that face loop and reverse loop have the same proportion in the Nos. 1-4, about 50%. These textiles show the same structures no matter what is the face or back side based on the double stitch knitting. The average thickness shows higher compared to the sample nos. 5–7 that consists of a different number of face stitches and reserve stitches. Texture no. 4 owns the largest repeat unit that its wale density is twice as large as no. 1. However, the number of face side units on the practical fabric is nearly a half decline than no. 1. This is because the more sinker loops are stretched with each other so that the column appearance can be formed. With the knitted unit increasing, the diameter of the column and the thickness of fabrics are enlarged, herein, decreasing the number of face side unit and the efficient contact area when triggering movements. In terms of rib structure with different proportions of face and reverse loop, the appearance exhibits the characteristic of single-faced structure obviously, with knitted repeat units increasing. Meanwhile, the wale density of no. 7 is as large as no. 1 and no. 5, but the number of face loop units has distinctive differences due to the number of the knitted unit is six loops that are much more than no. 1 (2 loops) and no. 5 (3 loops), so the output performance is lower than that of no. 1 and no. 5. As a result, the rib-knitted fabric no. 1 represents the most face loop units in nos. 1–10 during the contacting-separating movements.

**Table 2:** Parameters of knitted textiles

No.	The proportion between face side and reverse side/%	Wale density/in.	Course density/in.	Thickness/cm
no. 1	50	26	18	2.93
no. 2	50	32	18	2.25
no. 3	50	36	19	2.72
no. 4	50	42	19	3.36
no. 5	66.7	25	19	2.83
no. 6	40	30	19	3.07
no. 7	33.3	26	19	2.20
no. 8	-	18	36	2.71
no. 9	-	18	39	2.83
no. 10	-	18	10	3.01

On the other side, the shape-type knitted textile has been designed through the different number of loops assembling into the whole fabric, forming arch structures. Due to the direction of cord length is horizontal, the wale density of the fabric shows approximate stability in the transverse direction. The arch structure provides an approach for separating charge on the surface, which has a hollow inner space. Thus, the efficiency of harvesting wasted mechanism energy has been improved. Generally, in order to enhance output performance, an arch type is made of flexible materials with perfect elastic and durability, such as silicon substrates, but it is tough to be knitted in industrial knitting machine for meeting the commercial requirements. When it comes to see the arch structure is based on the knitted textile in previous researches [24, 41, 50], the construction needs to be sewed or taped, which is a complex and time-consuming process. We presented a knitted-arch textile that is prepared through the whole forming technique without second manufacturing that endows the high efficiency of production. Among the horizontal cord structures, the 0.3-cm height shows the lowest charge output compared to four needle and five needle horizontal cord structure with the height of 0.15 cm and 0.2 cm, respectively, which can be influenced by the low stiffness of knitted textiles in a large distance between both ends fixed. The highest convex shape is hard to keep arch with force pressure and recover to pristine shape, which leads to some charges are neutralized. As a result, the decrease of the arch height can enhance the tolerance of convex structures. However, such shape-type cords reduce the effective contact area which is a line type that has little areas than real contact, decreasing the performance of electrical-output.

**Fig. 5:**
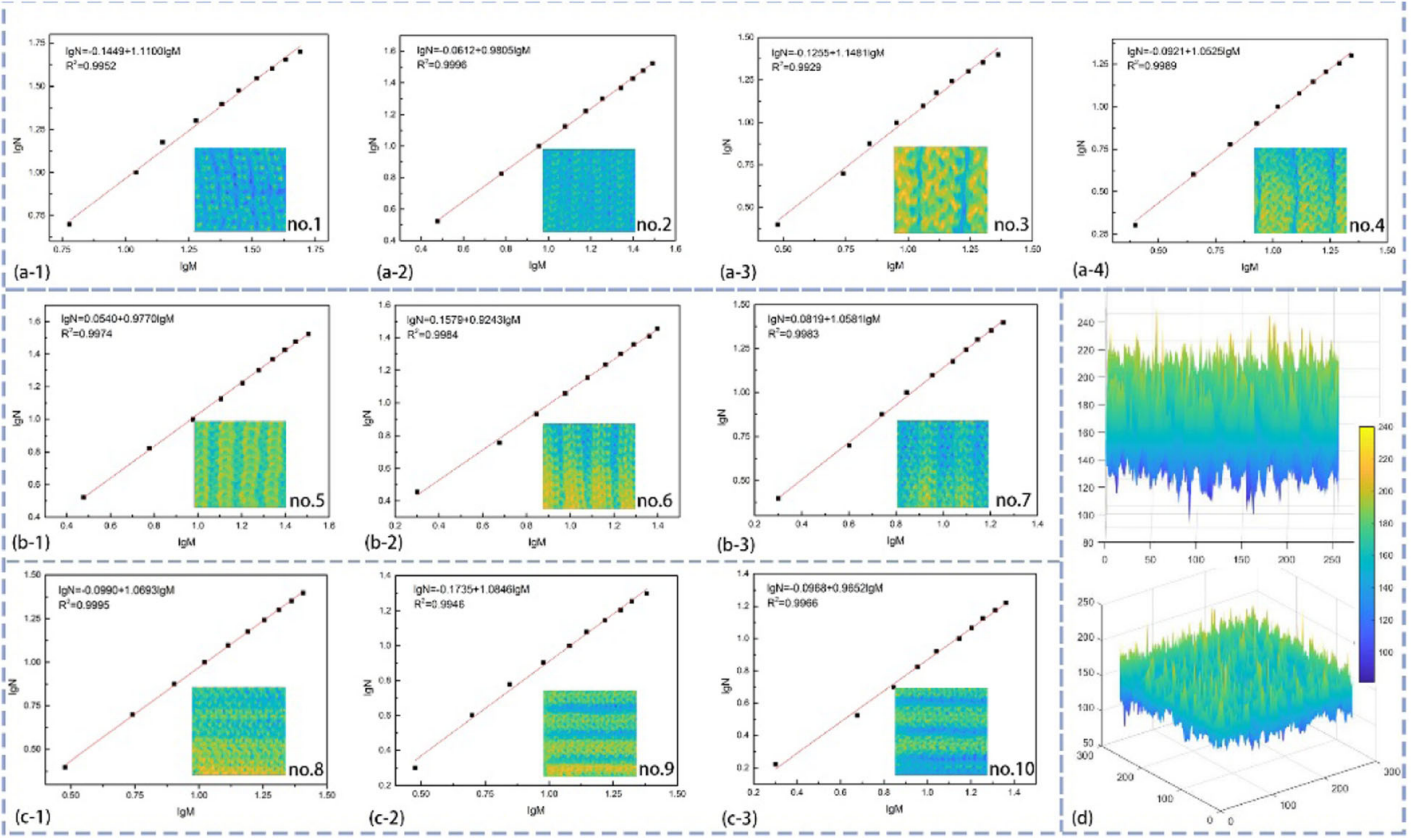
Fitting curve and some visual images for knitting textiles. **a** The m*n rib structure. **b** The 2*m rib structure. **c** Some needle horizontal cord structure. **d** The visual images from different aspects

Loops have irregular structures, so the evaluation of their geometry properties such as stitch size and surface shape is challenging. To identify the irregularity of the loops, traditional evaluation which is an integral dimension cannot be utilized. The fractal theory is suggested to analyze the category of irregularity in our surroundings and nature. The proposed concept of fractal dimension is an excellent tool for exhibiting complex morphology that presents the rules, the complexity, and the roughness of the textile surface. Because all fractals are not self-similar completely, the mathematical calculation is used to argue the geometry configuration. In order to understand the knitted structure’s surface, some images visualize the information carried in Fig. 5d. As shown in Fig. 5d, the characteristic of convex surface can be intuitively observed from different perspectives where the evidence for confirming the raised morphology is.

The uneven surface has been formed with the knit structure designed caused by the yarn morphology and structure design. The fractal geometry is an efficient calculation for evaluating the textile surface and understanding the characteristic of knitted structures and ability of triboelectric charge generation. In fact, with the increase of the raised unit, it can improve the uneven knitted textile owing to the surface shape modified. Although all of the knitted textile own convex structures in longitude and transverse direction, the degree of similarity is still not confirmed that is the significant reference value for whether using fractal dimension successfully or not. To estimate the feasibility of fractal dimension, all of the knitted fabrics are calculated through measuring the width of the convex unit, the size of loops in length, and width when textiles stay in stable size. Figure 5 a, b, and c show the fitting curve of fractal dimension of nos. 1–10 type fabrics, and slope of a line means the fractal dimension. The existence of the relationship is found in convex structures of the ten different types of knitted textiles, which confirms the fractal characteristic of ten knitted fabrics. Therefore, the fractal theory applied in the analysis of diversity knit structure that is practicable.

**Fig. 6:**
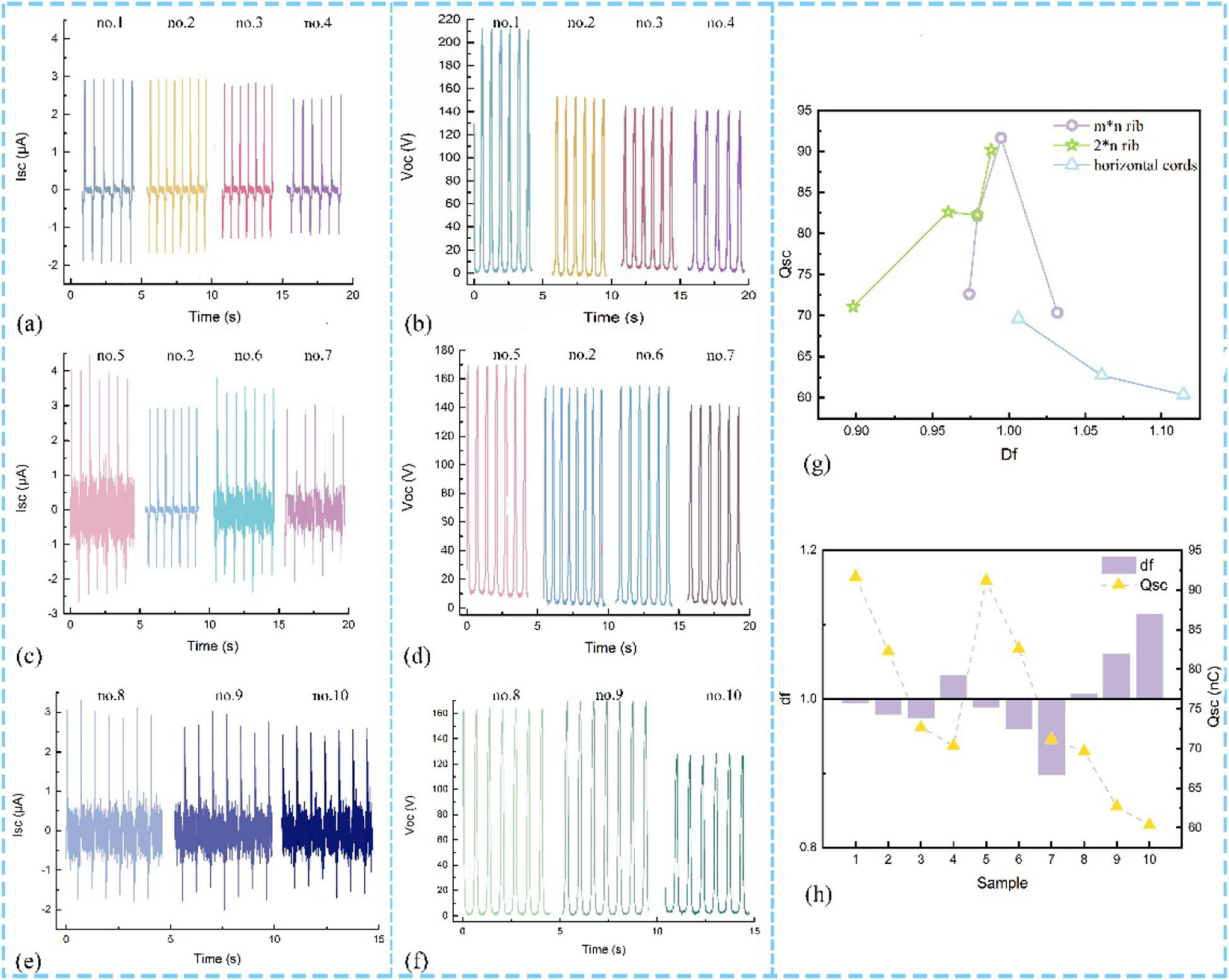
Schematic illustration of fractal dimension and generated Isc and Voc. **a** The Isc of m*n rib. **b** the Voc of the m*n rib. **c** The Isc of 2*m rib. **d** The Voc of the 2*m rib. **e** The Isc of n type. **f** The Voc of n type. **g** The D_f_-transfer charge curve. **h** The F value curve

Figure 6a–f illustrates the generated Isc and Voc based on the practical applications of contact and separation working KNGs, based on the structure types and shape types. There is a trend that a decrease with the knit unit increases about the Isc and Voc as shown in Fig. 6a–f. This is because the Isc is changed with the effective contact area which is affected by knit structures.

When calculating the *D*_f_ of various knit structures, the investigated knit structure states that the different knit structures have an unequal value which is non-integral dimension due to the different components of convex as demonstrated in Fig. 6g. As for Fig. 6g, this is the image of the transfer charge versus fractal dimension curve of diversity structures. The rib structure presents desirable output performance and the fractal dimension near the value of one. The TENGs based on structure-type knitted-textiles have a higher transfer charge than shape type and the value of *D*_f_ about the m*n rib type, 2*n rib type, and *n* type is in the range of 0–2, 0–1, and 1–2, respectively. Generally, the fractal dimension symbolizes the extent of surface roughness which is the roughness increasing with the large *D*_f_. However, the shape-type fabrics are designed in horizontal cord with small line-contact area, so the roughness has little influence on the transfer charge.

In order to demonstrate the influence on *D*_f_ of convex structure homogeneity in rib structures, the random side length is chosen and calculated. The result exhibits as follow:

1-4 $$ \varepsilon \left(a\ast b\right)=M\left(l\ast b\right) $$

1-5 $$ N=\frac{a}{l} $$

where *a* is the length of the whole fabric, *b* is the width of the convex unit and is equal to the width of the whole fabric, *l* is the length of the convex unit, *M* is the number of the convex unit, *N* is the repeated multiple of self-similar units that is the length of convex units to the length of whole samples, and *ε* is the proportion of face loop and reverse loop, meaning the uniform of the convex distraction.

Then, the calculation of *M* and *N* can be used in the formulation (1-3), the result shows that obtained *D*_f_ is not the same with the *D*_f_ that is calculated based on the length of actualmeasurement as shown in Table 3. No matter how the raised structure is distributed, the value of *D*_f_ is affected by the practical length and number of cords.

**Table 3:** Compared to *D*_f_ in random and actual length

No.	*D*_f_ random	D_f_ actual
no. 1	0.8488	0.9948
no. 2	0.8321	0.9793
no. 3	0.8190	0.974
no. 4	0.8168	1.0318
no. 5	1.1168	0.9884
no. 6	0.7784	0.9602
no. 7	0.7229	0.8979

It is noted that the fractal dimension of the 2*1 rib structure is close to the 1*1 rib reach at 0.99, and thus, the transfer charge is much the same as shown in Fig. 6g. The generated electrical-output performance shows the highest when the *D*_f_ is near the value of one. That has provided one guess if the fractal dimension can evaluate the surface morphology and character the output performance. To investigate the correlation of fractal and transfer charge, the difference between the fractal dimension and the value of one (named *F* value) has been illustrated in Fig. 6h. The operating results show a trend that is decreased *F* value can boost the much higher Voc, taking evidence for potential application of fractal dimension. However, the *F* value is regarded as an evaluation of the roughness structures, which needs to consider the properties of the primary loop of the structure. Then, the influence on transfer charge is discussed comprehensively. The sample of no. 4 and no. 6 has a similar F value, but the massive difference exists on both of output performance. The surface morphology of no. 4 shows the planar structure due to the same number of face and reverse loops, so the transfer charge is low. But no. 6 has prominent appearance due to the reverse loops over the face stitches and the generated large transfer charge when contacting and separating. Therefore, the selection and design of the knitted structure of the textile based on the *F* value highly improved the generated total electrical charge, which is an indispensable requirement for construct a high-effective flexible self-power device based on the knitted textiles.

## Conclusion

We have demonstrated that the knitted textile with high flexibility and excellent transfer charge can be applied in flexible TENGs for harvesting irregular and low-frequency biomechanical energy, which owns an outstanding output performance. To identify the relationship between surface morphology and output property, fractal theory has been used to quantify the surface geometry and used to evaluate its influence on the transfer charge ability of surface appearance. Different knit structures have been fabricated that can analyze their impact on energy harvesting. From the aspect of the knitted unit, the result shows that the maximum output of 1*1 rib structure can reach at 213 V with the minimum knitted unit. In addition, to further understand the working mechanism and the geometry of contact area, the various knit structures have been illustrated in a fractal dimension that is distinct from traditional dimension. Through calculation, different knitted structures with identical knit units can be used to obtain fractal dimension with the same knit units. The generated electrical output can be increased with the fractal dimension close to the value of one. Therefore, the difference between the fractal dimension and the value one can be used in the evaluation of transfer charge ability according to the irregular surface. In the near future, it is expected that an evaluation for generating output ability based on fractal theory in constructing a triboelectric nanogenerator, obtaining maximum output performance to optimize the flexible self-power system for harvesting wasted human motions in our daily life will be investigated.

## Data Availability

All data generated or analyzed during this study are included in this published article.

## Abbreviations

E-skins: Electronic skins
IoT: Internet of Things
PDMS: Polydimethylsiloxane
TENG: Triboelectric nanogenerator
PTFE: Polytetrafluoroethylene
D_f_: Fractal dimension
CS: Contact-separate working mode
KNG: The triboelectric nanogenerator based on knitted textile
Voc: The open-circuit voltage
Isc: Short-circuit current
Qsc: Transfer charge
F value: The difference between the fractal dimension and the value of one
